# First record of *Taenia krabbei* Moniez, 1879, muscle cysts in roe deer (*Capreolus capreolus*) in Carinthia, Austria

**DOI:** 10.1016/j.ijppaw.2026.101247

**Published:** 2026-06-07

**Authors:** Jutta Pikalo, Michael Dieter Mansfeld, Wolfgang Geier, Ivan Stimac, René Brunthaler, Josef Harl, Gunther Vogl, Hans-Peter Fuehrer

**Affiliations:** a Institute of Parasitology, Department of Biological Sciences and Pathobiology, University of Veterinary Medicine Vienna, Veterinärplatz 1, Vienna, 1210, Austria; b Carinthian Institute for Food Safety, Veterinary Medicine and Environment, Kirchengasse 43, Klagenfurt am Wörthersee, 9020, Austria; c Medical Practice Dr. Geier, Tobis 100, Preding, 8504, Austria; d Institute of Pathology, Department of Biological Sciences and Pathobiology, University of Veterinary Medicine Vienna, Veterinärplatz 1, Vienna, 1210, Austria

**Keywords:** *Taenia krabbei*, Tapeworm fins, *Cysticercus tarandi*, Molecular biology, Histopathology, Wildlife parasitology, Food safety

## Abstract

The present report describes the first record of the tapeworm *Taenia krabbei* Moniez, 1879, represented by its larval stage, in roe deer (*Capreolus capreolus*) from two hunting districts in Carinthia, Austria. The cysticerci of *T*. *krabbei* were found during gross meat inspection and isolated from the muscle. The specific identification was based on molecular analysis of a partial sequence of the mitochondrial cytochrome *c* oxidase subunit I (*COI*) gene. The definitive host was not identified in this report. Grey wolves (*Canis lupus lupus*) are suspected to be the definitive host, as they are reported to be resettling in the area. The finding of muscle cysts of *T*. *krabbei* raises concerns for hunters and producers of venison, as affected carcasses are often rejected based on visual inspection criteria. Consequently, systematic assessment of the distribution of *T. krabbei* across both intermediate and definitive host populations is warranted. In parallel, targeted sanitary education initiatives for hunters are essential to discourage practices that may facilitate transmission and perpetuation of the parasite's life cycle.

## Highlights

• First report of *Taenia krabbei* muscle cysts in seven roe deer from Austria.
• Detected by routine meat inspection.
• Overall prevalence of approximately 9% across the two hunting districts.
• Confirmed by *COI* sequencing and phylogenetic analysis.

## Graphical abstract

**Figure:**
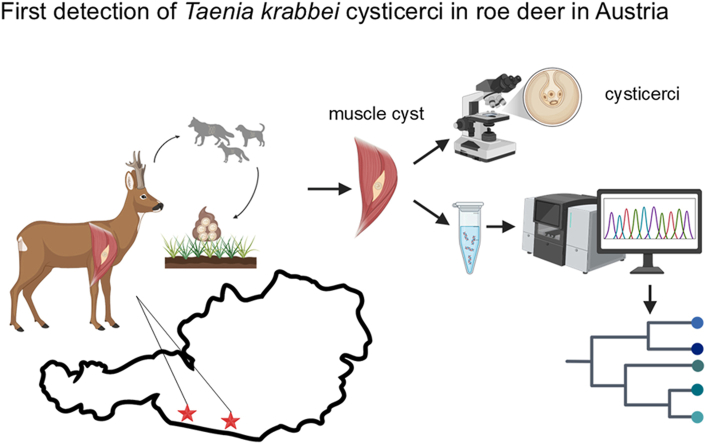

## Abbreviations

**bp**: base pair
***COI***: mitochondrial cytochrome *c* oxidase subunit I

**CI** Confidence interval

**HE** hematoxylin–eosin

### 1 Introduction

The cestode *Taenia krabbei* Moniez, 1879 is a tapeworm of the family Taeniidae Ludwig, 1886, with a sylvatic life cycle involving canids (mainly grey wolves (*Canis lupus lupus*), but also red foxes (*Vulpes vulpes*), domestic dogs (*Canis lupus familiaris*), and other canids) as definitive hosts, and cervids (various ungulates, including roe deer (*Capreolus capreolus*), fallow deer (*Dama dama*), red deer (*Cervus elaphus*), moose (*Alces alces*), elk (*Cervus canadensis*), and reindeer (*Rangifer tarandus*)) as intermediate hosts (Kolár et al., 1978; Gibbs and Eaton, 1983; Jones and Pybus, 2001; Priemer et al., 2002; Flueck and Jones, 2006; Lavikainen et al., 2011; Al-Sabi et al., 2013; Formenti et al., 2018; Siben and Nikonov 2022; Suleimenov et al., 2026). Wolves, dogs, foxes, and other canids become infected by ingesting larval stages present in the tissues of infected intermediate hosts. Following ingestion, the tapeworm develops in the small intestine and produces eggs that are subsequently shed in the feces. In definitive and intermediate hosts, infection is typically asymptomatic. The intermediate hosts are infected via ingestion of eggs present in contaminated feed or water. After ingestion, the eggs develop into larva that migrate into the muscles, where they form small cysts containing the larval stage (metacestodes, cysticercus, *Cysticercus tarandi*). Cysticerci typically develop in the striated muscles and cardiac tissues of cervids, reflecting a long-established parasitic relationship across boreal and temperate ecosystems of the Northern Hemisphere (Europe, North America, and Asia) (Jones and Pybus, 2001). In some cases, infection of the intermediate hosts may lead to clinical signs such as weakness or inflammatory processes (Al-Sabi et al., 2013; Siben and Nikonov, 2022). The presence of *T. krabbei* has been documented in several countries, including Finland, Sweden, Norway, Poland, Germany, Denmark (including Greenland), the Czech Republic, Italy, France, Romania, the United States of America, Canada, Southern Kazakhstan, and Russia (Kolár et al., 1978; Gibbs and Eaton, 1983; Duffy et al., 1994; Kapel and Nansen, 1996; Priemer et al., 2002; Stien et al., 2010; Raundrup et al., 2012; Al-Sabi et al., 2013, 2018; Gori et al., 2015; Poglayen et al., 2017; Formenti et al., 2018; Bindke et al., 2019; Citterio et al., 2021; Juránková et al., 2021; Siben and Nikonov, 2022; Umhang et al., 2023; Moraru et al., 2026; Suleimenov et al., 2026).

Historically, reports of *T. krabbei* muscle cysts in roe deer (*Capreolus capreolus*) have been rare, with early descriptions limited to incidental findings in other cervid species (e.g., reindeer and elk) or in geographically distant regions (Siben and Nikonov, 2022). Cysticerci of *T*. *krabbei* in roe deer have been reported in Denmark, the Czech Republic and Italy (Kolár et al., 1978; Al-Sabi et al., 2013; Formenti et al., 2018).

Although *T. krabbei* is not considered zoonotic, infection can lead to the condemnation of carcasses for aesthetic reasons, and poses implications for wildlife health assessments, game meat hygiene, and the ecology of host–parasite interactions in wild ungulate populations (Jones and Pybus, 2001; Raundrup et al., 2012).

In the current report we describe an infection with cysticerci of *T*. *krabbei* in the muscle of roe deer (*Capreolus capreolus*) in two different hunting districts in Carinthia, Austria.

### 2 Materials and methods

#### 2.1 Origin of the roe deer

**Fig. 1:**
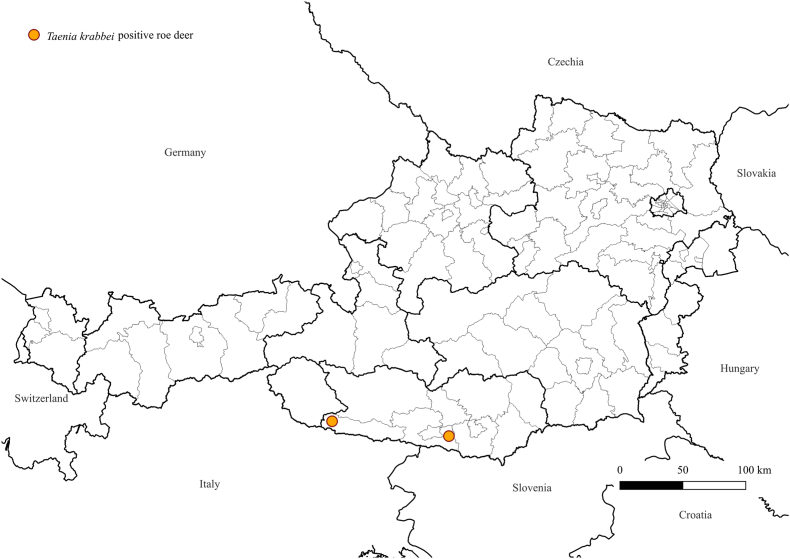
Location of the *T*. *krabbei*-positive roe deer in Carinthia, Austria. Created with QGIS version 3.44.6-Solothurn (qgis.org).

From August to October 2025, in total 81 roe deer were shot by hunters in two different hunting districts, Lesachtal (70 animals, six animals had muscle cysts) and upper Rosental (eleven animals shot, one animal had muscle cysts), within a distance of more than 100 km in Carinthia, Austria (Fig. 1).

During butchering of the game, numerous cysts were observed in the muscle tissue. The meat was declared unsuitable for human consumption. Muscle tissue samples with individual cysts were preserved in 70% ethanol and sent to the office of the Carinthian Institute for Food Safety, Veterinary Medicine and Environment, and the Institute of Parasitology, University of Veterinary Medicine Vienna, for further identification.

#### 2.2 Parasitological examination

The muscle samples were analysed macroscopically by visual examination, and microscopically with a Stereomicroscope (SMZ1270; Nikon, Vienna, Austria) under 40 x magnification. Subsections were formalin-fixed, paraffin wax embedded, and further processed to 3 μm thin sections. Sections were stained with hematoxylin–eosin (HE) to evaluate the tissue and cellular architecture.

#### 2.3 Molecular- and phylogenetic analysis

For molecular analysis one to two muscle cysts per roe deer were opened and after removal from the meat sample, each larva was placed separately into a 2 ml microcentrifuge tube, along with one 3 mm tungsten carbide bead (QIAGEN, Hilden, Germany) and 500 μl ultrapure water. In total, one to two larva per animal were analysed separately, depending on the number of samples submitted. The samples were homogenized for 10 min at 30 Hz using a Tissuelyser II (QIAGEN, Hilden, Germany). The tubes were centrifuged for 10 s at 15.860 x g with an Eppendorf 5424 (Eppendorf, Vienna, Austria) and the supernatant (∼470 μl) transferred into a fresh 1.5 ml microcentrifuge tube, to which 180 μl ATL buffer and 20 μl Proteinase K (QIAGEN, Hilden, Germany) were added, vortexed and incubated overnight at 56 °C and 400 rpm (ThermoMixer F1.5; Eppendorf, Vienna Austria). DNA was extracted separately of each lysed sample (∼670 μl) using the IndiMag Pathogen Kit on the IndiMag 48s platform (Indical, Leipzig, Germany) according to manufacturer's instructions. Negative control was included to monitor potential contamination. All DNA extracts were stored at −80 °C until further use.

To determine the species of *Taenia*, the samples were subjected to DNA barcoding. A 396 bp fragment of the mitochondrial cytochrome *c* oxidase subunit I (*COI*) gene was amplified by standard PCR using the following primer pair:

COI_Bowles_Fw (for): 5′- TTT TTT GGG CAT CCT GAG GTT TAT -3′ and COI_Bowles_Rv (rev): 5′- TAA AGA AAG AAC ATA ATG AAA ATG -3’ (Bowles et al., 1992)

The master mix contained a final volume of 25 μl, consisting of 12.4 μl RNase-free water, 5 μl green reaction buffer (Promega, Walldorf, Germany), 0.5 μl PCR nucleotide mix 25 mM at 0.5 mM final concentration (Promega, Walldorf, Germany), 2.5 μl of each primer at 1 μM final concentration (Microsynth Vienna, Austria), 0.12 μl GoTaq G2 DNA Polymerase (Promega, Walldorf, Germany) and 2.5 μl DNA template. For each PCR run a negative and positive control was included. Genomic DNA from a positive sample, which was previously confirmed by sequencing, was used as a positive control. The PCR reaction was performed using the Biometra TOne thermocycler by Analytik Jena (Analytik Jena, Göttingen, Germany) under the following PCR conditions: 2 min at 95 °C; 40 cycles of 45 s at 95 °C, 45 s at 53 °C, 90 s at 72 °C; followed by 5 min at 72 °C. The PCR products were visualized using 1.8% agarose gel electrophoresis stained with Midori Green Advanced dye (Biozym Scientific, Hessisch Oldendorf, Germany). Samples that yielded a PCR product of the expected amplicon size were analysed by Sanger sequencing using the amplification primers at an external commercial laboratory (Microsynth, Vienna, Austria). The resulting *COI* sequences were assembled using BioEdit version 7.7.1 (North Carolina State University, Raleigh, USA) and subjected to a BLAST search on NCBI GenBank (National Center for Biotechnology).

A Median-Joining haplotype network was calculated based on the *T. krabbei COI* sequences covering nucleotide positions 733–1128 from NCBI GenBank and the present study. A BLAST search on GenBank retrieved 52 *T. krabbei COI* sequences. The sequences were aligned with MAFFT v.7.471 (Katoh and Standley, 2013) using the default options and trimmed to the length of the shortest sequence contained in the alignment. Three sequences with ambiguity characters (JF261324, KY012310, PV857611) were excluded. The final alignment had a length of 325 bp and contained 50 sequences. The Median-Joining haplotype network was calculated with Network 10.2.0.0 (Fluxus Technology Ltd, Suffolk, UK) using the default settings. The network was graphically arranged and provided with information on host species and countries with Network Publisher v.2.1.2.5 (Fluxus Technology Ltd) and edited with Adobe Illustrator CC v.2015 (Adobe Inc., San José, CA, USA).

### 3 Results

#### 3.1 Prevalence

Between August and October 2025, a total of 81 roe deer were shot in two hunting districts in Carinthia, Austria. In the Lesachtal district, muscle cysts were detected in six of 70 animals (8.6%), whereas one of eleven animals (9.1%) examined in the Upper Rosental district was positive for muscle cysts. The overall prevalence of *T*. *krabbei* was 8.6% (CI_95%_ = 2.5-14.8%).

#### 3.2 Macroscopic analysis

**Fig. 2:**
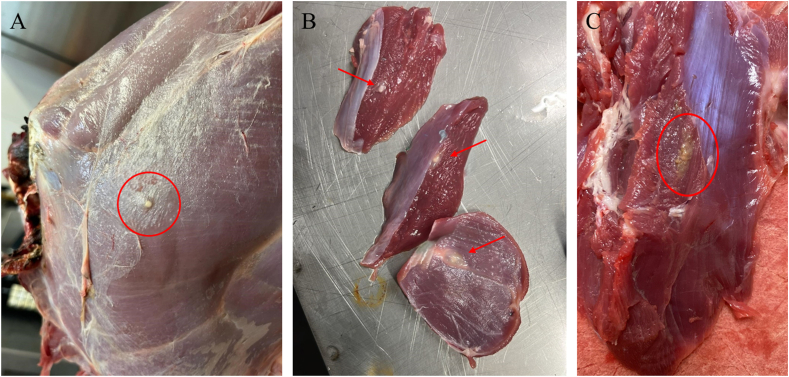
Cysts in roe deer meat. (**A**) Cyst (5 mm) in roe deer game meat; (**B**) Cyst in muscle tissue from roe deer. In both figures (**A, B**), the cysts appear as elongated, semi-transparent, fluid-filled sacs containing the larva of *T*. *krabbei*, with a distinct white spot (“head”) visible at one end; (**C**) Degenerated cyst in muscle tissue from roe deer.

Macroscopic examination of the submitted roe deer muscle samples (one to three per animal from in total seven roe deer) revealed different developmental stages with numerous white muscle cysts measuring approximately 2–6 mm in diameter (Fig. 2A and B). Degenerated muscle cysts have been detected as caseous or calcified lesions within the skeletal musculature (Fig. 2C).

#### 3.3 Microscopical analysis and histopathology

**Fig. 3:**
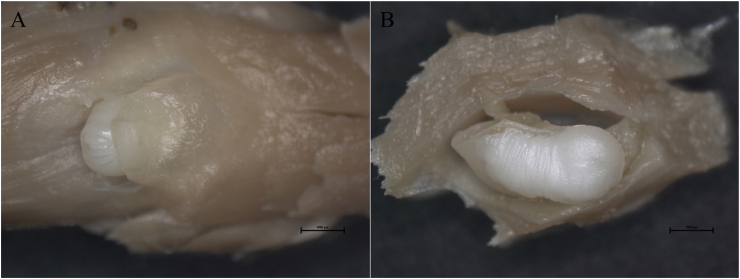
Microscopic characteristics of the muscle cyst and from the larva of *T*. *krabbei*. (**A**) Macroscopic appearance of an open muscle cyst (2–6 mm) with the membrane in skeletal muscle, revealing the larva of *T*. *krabbei*; (**B**) Macroscopic view of an opened muscle cyst presenting a small, round to oval, bladder surrounded by a connective tissue capsule or sequestrum. Each muscle cyst contains only a single larva. Bar = 1 mm.

During microscopic analysis, muscle cysts containing a single larva were observed (Fig. 3A and B). The cysts consisted of a thin cyst wall enclosing parenchymal tissue. Characteristic scolex structures were identified within the larva. The cysticerci were small (2-6 mm), round to oval, fluid-filled bladders. Each larva were surrounded by a connective tissue capsule or a sequestrum.

**Fig. 4:**
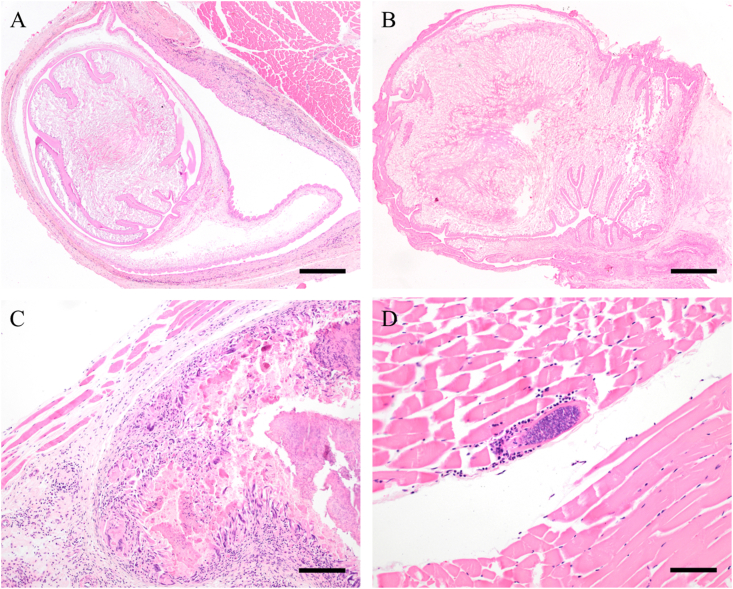
Histopathological characteristics of the cysticercus. (**A**) Cysticercus with a bladder membrane and with larval scolex. The endomysium and perimysium of the muscle may be expanded by an eosinophilic fibrillary (collagen) matrix. Bar = 400 μm; (**B**) Cysticercus of *T*. *krabbei*. Bar = 400 μm; (**C**) Severe inflammatory reaction, with infiltration of eosinophils, lymphocytes, macrophages, and multinucleated giant cells. Bar = 160 μm; (**D**) A cyst located within the skeletal muscle that shows no or only very slight accompanying inflammatory reaction. Bar = 80 μm.

Histopathological examination of embedded muscle samples showed a cysticercus with a bladder membrane and watery, transparent fluid (Fig. 4A and B). The encapsulation of the cysticercus varied from a thin, non-cellular collagen layer to a granulomatous inflammation with large, foreign body giant cells (Fig. 4C). Apart from that, no inflammatory reaction was observed in the muscles, but low to high numbers of cysts were noticed (Fig. 4D).

#### 3.4 Molecular- and phylogenetic analysis

One mt *COI* sequence was obtained from a larva from the muscle cyst of each animal, resulting in seven sequences that were 100% identical and one sequence was uploaded to GenBank (PZ120873).

**Fig. 5:**
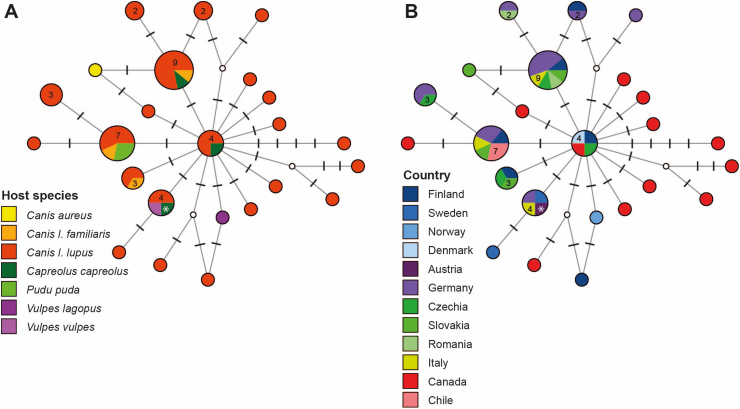
Median-Joining DNA haplotype network of partial (325 bp) *COI* sequences belonging to *T*. *krabbei*. The two figures show the distribution in (**A**) host species and (**B**) countries. Each circle represents a unique haplotype/lineage. The frequency is indicated for all haplotypes with more than one record and roughly corresponds to the size of circles. Bars on branches indicate the number of substitutions between two haplotypes. Small white circles represent median vectors, which are hypothetical (often ancestral or unsampled) sequences required to connect existing haplotypes with maximum parsimony. The lineages analysed in the present study are marked with asterisks.

The Median-Joining haplotype network calculated based on the alignment containing 50 *T. krabbei COI* sequences (325 bp) featured 24 haplotypes, which were found in wolves, dogs, golden jackals (*Canis aureus*), red foxes (*Vulpes vulpes*), polar foxes (*Vulpes lagopus*), southern pudus (*Pudu puda*), and roe deer (Fig. 5). The haplotypes differed by a maximum of 7 bp (2.2%) from each other. Most haplotypes were connected to a central haplotype that was found in wolves in Canada (KX058189), Czechia (MT227288), and Finland (JF261323), and in roe deer in Denmark (JX560319). The haplotype found in the present study was previously detected in wolves in Germany (KY012311) and Sweden (JF261327), and a red fox in Italy (MT806361). However, in the complete 396 bp fragment, only the sequence obtained from a wolf in Sweden (JF261327) was identical.

### 4 Discussion

The present report represents the first documented occurrence of *T*. *krabbei* muscle cysts in roe deer (*Capreolus capreolus*) in Carinthia, Austria, expanding the known geographic distribution of this parasite in cervid intermediate hosts.

The increasing abundance of cervids and the ongoing recovery and expansion of wolf populations may facilitate parasite transmission in alpine ecosystems. This is particularly relevant for Austria, where the current wolf population is largely sustained by dispersing individuals originating from the Italian Alpine and Central European populations (Kaczensky et al., 2024; Austrian Center for Bear, Wolf, and Lynx, 2026). Consequently, the natural migration and recolonization of wolves from Italy into the Austrian Alps (Kaczensky et al., 2024; Austrian Center for Bear, Wolf, and Lynx, 2026) may contribute to the spread and maintenance of the *T*. *krabbei* life cycle across the transboundary Alpine region, particularly given that *T*. *krabbei* has already been documented in both wolves and roe deer in Italy (Gori et al., 2015; Poglayen et al., 2017; Formenti et al., 2018).

Historically, *T. krabbei* muscle cysts have been considered uncommon in roe deer, with the most frequently cited modern case being the reappearance of *T. krabbei* muscle cysts in a roe deer from Denmark after more than six decades (Al-Sabi et al., 2013).

Prior to these recent findings, *T. krabbei* had been sporadically recorded in other ungulate hosts, such as red deer and moose, with additional records from Greenland muskoxen (*Ovibos moschatus*), caribou (*Rangifer tarandus*), mouflon (*Ovis musimon*), and the latest report from southern pudu deer (*Pudu puda*), pointing to a broader host range under specific ecological conditions (Clausen et al., 1980; Jones and Pybus, 2001; Raundrup et al., 2012; Marimán et al., 2026).

Furthermore, the first molecular confirmation of *T. krabbei* in roe deer and a sympatric semi-stray dog in northern Italy underscores the parasite's capacity to circulate at the wildlife–domestic interface and suggests that host overlap among wild cervids and canids may facilitate transmission (Formenti et al., 2018). Especially working dogs, particularly those used for hunting or herding, may become infected and play a role in the transmission of the pathogen (Formenti et al., 2018). These findings emphasize that the emergence of *T. krabbei* infections in roe deer may reflect a novel introduction, particularly in areas where canid definitive hosts (e.g. grey wolves, red foxes, and dogs) prey upon cervids or scavenge infested carcasses. In this study, the introduction of *T*. *krabbei* is most likely associated with grey wolves, which represent the most probable source, as they frequently prey on roe deer and red deer and harbour *T*. *krabbei* as their most common helminth species (Flühr, 2011; Lesniak et al., 2017; Juránková et al., 2021). In North and East Germany, a slightly higher prevalence of muscle cysts of *T*. *krabbei* was found in ungulates originating from wolf-inhabited areas compared to control regions (Lesniak et al., 2017). This pattern suggests that the prevalence of wolf-associated parasites declined during periods of wolf absence and has increased again following recolonization (Lesniak et al., 2017). Though, the involvement of other canid definitive hosts in the investigated parasite life cycles cannot be excluded. The definitive host could not be identified in this study.

Definitive host ecology is a key determinant of *T. krabbei* transmission dynamics. Grey wolves, red foxes, and domestic dogs are known as definitive hosts for several *Taenia* species, and their feeding habits directly influence the parasite's life cycle by providing access to infected intermediate host tissues (e.g., cysticerci in cervid muscle) (Formenti et al., 2018; Schneider et al., 2024). In addition to the well-known definitive hosts of *T*. *krabbei* (wolves, foxes, and dogs), recent reports from Arctic foxes (*Vulpes lagopus*), golden jackals (*Canis aureus*), brown and black bears (*Ursus arctos*, *Ursus americanus*), and Eurasian lynx (*Lynx lynx*) further expand the known definitive host range (Duffy et al., 1994; Kapel and Nansen, 1996; Jones and Pybus, 2001; Stien et al., 2010; Kołodziej-Sobocińska et al., 2018; Suleimenov et al., 2026).

In Austria, the distribution and density of wild canids, particularly wolves, but also lynx and golden jackals, have increased in recent years (Blaschka and Rauer, 2021), potentially elevating contact rates between predators and roe deer. According to the Austrian Center for Bear, Wolf, and Lynx (2026), there are four wolf packs in Carinthia: three located in the Hermagor district and one in the Völkermarkt district. All roe deer testing positive for *T*. *krabbei*, with the exception of the individual from Rosental, originate from the Hermagor district. In the area of Rosental, individual wolves have been observed (Austrian Center for Bear, Wolf, Lynx, 2026). Given the current recolonization dynamics in the Eastern Alps, wolves occurring in Carinthia are presumed to originate from neighboring Italy and to belong to the Alpine population, where *T*. *krabbei* has already been documented in the animals (Gori et al., 2015; Poglayen et al., 2017; Formenti et al., 2018; Kaczensky et al., 2024; Austrian Center for Bear, Wolf, and Lynx, 2026). Such shifts in predator–prey dynamics may facilitate the maintenance and spread of *T. krabbei* within wild ungulate populations, although further quantitative data on prevalence and predator diet composition are needed to substantiate this hypothesis.

Diagnostic methodology also plays a crucial role in recognizing and reporting *T. krabbei* infections. Traditional postmortem inspection often identifies macroscopic cysts. However, morphological features alone are insufficient for species-level identification due to similarities among taeniid cysticerci. A morphological identification is not possible when the cysticerci and the larval scolex are not yet fully developed, as was the case in the present study. Accurate determination of the size of the scolex and the rostellar hooks is only possible after their complete development, which occurs around 56 days after infection. On the other hand, a large proportion of *T. krabbei* muscle cysts are eliminated by the host during early developmental stages (4 to 8 weeks of maturation, after which they may become calcified), including the differentiating embryo and before rostellar hooks have begun to develop, whereas only a small number are destroyed once the cyst has fully developed (Sweatman and Henshall, 1962). In the present study, some calcified cysts and capsules were also observed, indicating a subacute or chronic infection, that had likely persisted for more than two months (Sweatman and Henshall, 1962). In contrast, larva in the cyst can live in the host tissue for several years (Sweatman and Henshall, 1962). Molecular techniques, particularly mitochondrial gene sequencing (e.g., *COI*), provide reliable identification and have become essential for distinguishing *T. krabbei* from other taeniid species in wildlife surveys (Al-Sabi et al., 2013; Formenti et al., 2018). Therefore, a broader range of genetic loci and isolates from diverse geographic regions and different hosts should be examined for accurate characterization of the population genetics of this species (Lavikainen et al., 2011; Formenti et al., 2018). The integration of molecular diagnostics in routine wildlife health monitoring would likely enhance detection and improve epidemiological insights.

Although *T*. *krabbei* is not considered a direct public health risk, its occurrence in game species has important implications for meat hygiene and the economic value of hunting. Muscle cysts may lead to carcass condemnation for aesthetic reasons, thereby reducing the marketability of harvested game (Jones and Pybus, 2001; Hoberg, 2002; Formenti et al., 2018). The infected meat should also not be fed to dogs, as they can acquire the infection through the larval stages. Altered musculature should not be left at feeding or scavenging sites, in order to interrupt the parasite's life cycle (Formenti et al., 2018). This finding underscores the need for awareness among hunters and wildlife managers regarding parasite prevalence, the lifecycle of *T*. *krabbei*, and its potential effects on game meat quality. While meat containing *T. krabbei* muscle cysts may appear visually unappealing, the adult parasite is unable to establish infection in humans and has never been reported as zoonotic (Palmer et al., 1998; Jones and Pybus, 2001; Deplazes et al., 2019). Consequently, in Austria, local hunters are not prohibited from selling infested game meat at open markets. Nevertheless, other tissue-dwelling parasites may pose genuine food safety concerns, and accurate differential diagnosis can be challenging (Raundrup et al., 2012; Formenti et al., 2018).

### 5 Conclusion

In conclusion, this study documents the first confirmed occurrence of *T*. *krabbei* in roe deer from Carinthia, Austria, thereby expanding the known geographic range of this parasite. Although the definitive host could not be conclusively identified, the potential involvement of wolves highlights the need for further ecological investigations into predator–prey dynamics and parasite transmission pathways in the region. The detection of cysticerci during routine meat inspection has direct economic implications for hunters and venison producers due to carcass condemnation. Therefore, comprehensive surveillance programs targeting both intermediate and definitive hosts are essential to assess the true distribution and epidemiological significance of *T. krabbei*. In parallel, the implementation of targeted sanitary education for hunters is critical to mitigate behaviors that may contribute to the maintenance and spread of the parasite's life cycle.

### Informed consent statement

Informed consent for sampling and publication was obtained from the hunters involved in the study.

### Availability of data and materials

The authors confirm that all data underlying the findings in this work are fully available and are presented herein.

### Ethics approval and consent to participate

Roe deer are not a protected species in Austria and may be shot by licensed hunters outside the closed season without a special permit. No animals were killed specifically for this study. The samples were taken from dead animals during the hunting season. As the project did not involve live animals or animal experiments, nor did it involve sensitive patient data, the study did not have to be reported to the Ethics and Animal Welfare Committee (ETK) according to point 1.2 and 1.3 of the guidelines regarding Good Scientific Practice (Ethics in Science and Research) of the University of Veterinary Medicine Vienna.

### Institutional review board statement

Not applicable.

### Funding

This research did not receive any specific grant from funding agencies in the public, commercial, or not-for-profit sectors. This work has been funded for publication by the 10.13039/501100009088University of Veterinary Medicine Vienna.

### CRediT authorship contribution statement

**Jutta Pikalo:** Conceptualization, Data curation, Formal analysis, Investigation, Methodology, Project administration, Supervision, Visualization, Writing – original draft, Writing – review & editing. **Michael Dieter Mansfeld:** Data curation, Investigation, Methodology, Writing – review & editing. **Wolfgang Geier:** Data curation, Investigation, Writing – review & editing. **Ivan Stimac:** Data curation, Investigation, Methodology, Visualization, Writing – review & editing. **René Brunthaler:** Data curation, Investigation, Methodology, Visualization, Writing – review & editing. **Josef Harl:** Formal analysis, Investigation, Methodology, Writing – review & editing. **Gunther Vogl:** Writing – review & editing. **Hans-Peter Fuehrer:** Project administration, Resources, Supervision, Writing – review & editing.

## Conflicts of interest

The authors declare no conflict of interest.
